# Sharing health-related data: a privacy test?

**DOI:** 10.1038/npjgenmed.2016.24

**Published:** 2016-08-17

**Authors:** Stephanie OM Dyke, Edward S Dove, Bartha M Knoppers

**Affiliations:** 1 Centre of Genomics and Policy, Department of Human Genetics, Faculty of Medicine, McGill University, Montreal, QC, Canada; 2 JK Mason Institute for Medicine, Life Sciences and the Law, School of Law, University of Edinburgh, Edinburgh, UK

## Abstract

Greater sharing of potentially sensitive data raises important ethical, legal and social issues (ELSI), which risk hindering and even preventing useful data sharing if not properly addressed. One such important issue is respecting the privacy-related interests of individuals whose data are used in genomic research and clinical care. As part of the Global Alliance for Genomics and Health (GA4GH), we examined the ELSI status of health-related data that are typically considered ‘sensitive’ in international policy and data protection laws. We propose that ‘tiered protection’ of such data could be implemented in contexts such as that of the GA4GH Beacon Project to facilitate responsible data sharing. To this end, we discuss a Data Sharing Privacy Test developed to distinguish degrees of sensitivity within categories of data recognised as ‘sensitive’. Based on this, we propose guidance for determining the level of protection when sharing genomic and health-related data for the Beacon Project and in other international data sharing initiatives.

## Introduction

The Global Alliance for Genomics and Health (Global Alliance^1^) is an international coalition dedicated to improving human health by maximising the potential of genomic medicine through data sharing. Central to this endeavour is its *Framework for Responsible Sharing of Genomic and Health-Related Data.*^2^ While the broad benefits of such sharing are largely uncontested, greater access to potentially sensitive data raises important ethical, legal and social issues (ELSI).^3,4^ If not properly addressed, these issues risk hindering and even preventing useful data sharing that can improve human health. One such important issue is respecting the privacy-related interests of individuals whose data are used in genomic research and clinical care.^5^

In all kinds of data sharing—publishing it freely on the world wide web or sharing it only with approved users or under strict controls—the question of which data present higher privacy risk, and therefore warrant higher protection, arises. We have aimed to determine which types of genomic and health data would require either a lower or higher level of protection in an ethically and legally coherent manner, and to enable this classification, developed a Data Sharing Privacy Test. We drew on the legal term ‘sensitive data’ in data protection and privacy regulation around the world to identify all health-related data categories that may occur in practice and would likely require higher protection, at least for some types of information in these categories (e.g., personal data concerning sexually-transmitted disease). As the recently agreed EU General Data Protection Regulation notes: ‘Personal data which are, by their nature, particularly sensitive in relation to fundamental rights and freedoms, deserve specific protection as the context of their processing may create important risks for the fundamental rights and freedoms’.^6^ At the same time, however, the Regulation acknowledges that an absolute prohibition on processing (including sharing) sensitive categories of data is not realistic. Processing should be allowed when subject to suitable safeguards so as to protect fundamental rights. This permission is particularly noted where grounds of public interest justify, including for the prevention or control of communicable diseases and other serious threats to health, and scientific research purposes.

The purpose of the proposed Data Sharing Privacy Test is to determine which types of information in these sensitive data categories would fall within or outside a zone of heightened protection. In proposing a novel method to implement ‘tiered protection’ of data, this approach may simultaneously allow us to determine which information will only entail *de minimis* risk in certain contexts such as the Beacon Project, which we used as a test case, and so be largely exempt from additional protection. The Beacon Project is a key Global Alliance Demonstration Project that was launched as a test of the willingness of international research and clinical sites to share genomic data in the simplest of all technical contexts (ga4gh.org/#/beacon). Its goal is to address barriers to international genomic data sharing by fostering the creation and development of ‘Beacons’ that share data in a manner that respects the interests of research participants, including their privacy. A Beacon is a simple public web service that any institution can implement, designed to accept simple queries such as ‘Do you have record of any genomes with an 'A' at position 100,735 on chromosome 3'. If Beacon databases are large enough that inclusion in them does not in itself reveal anything ‘sensitive’, and responses to Beacon queries do not provide individually identifying information and are considered with respect to data sensitivity, privacy is not an issue.

We believe the Data Sharing Privacy Test to be widely applicable to genomic research and clinical data sets of use in both research and clinical care. We also provide additional guidance in the form of points-to-consider to facilitate consistent assessment of privacy risk.

## Results

### Categories of sensitive data

How we categorize and classify sensitive data (i.e., what factors influence judgments about the degree of sensitivity) is an ongoing critical challenge, as what makes something (and who determines) ‘sensitive’ is highly context-dependent. Sensitive data, or ‘sensitive information’ as may be synonymously termed in law, is a vague concept of varying interpretation in data privacy legislation, administrative treatment and case law around the world. In data privacy law, sensitive data receive heightened levels of data protection beyond the baseline protections otherwise required. Typically, individuals and organisations may only collect, store, or otherwise process sensitive data for specific purposes and under special conditions, such as the explicit consent of the data subject (though this latter condition is sometimes subject to certain exceptions, such as scientific research purposes with proper governance oversight). Furthermore, for our purposes, defining categories of health-related data requiring an assessment of data sensitivity in the context of genomic data sharing for research and clinical care entailed going beyond analysis of the defined (and rigid) sensitive data categories in data privacy legislation so as to identify other forms of personal data that may be present in relevant data sets and receive heightened data privacy protection under the law. For example, telecommunication providers are often required to give heightened data protection to customers’ geolocation data associated with their mobile phones, as these data can reveal precise information about the physical location of users. These protections typically include obtaining the customer’s explicit consent before disclosing this information to other businesses for marketing purposes. Second, as most of the data privacy legislation we reviewed is designed to apply much more broadly than to data in the healthcare or research sector, selecting potential categories of sensitive data involved assessing how general categories of sensitive data (e.g., a person’s religious views) might intersect with genomic and associated health data used in research and clinical care.

We propose that several categories of health-related data warrant careful consideration for the purposes of implementing responsible data sharing practices (Table 1). While there may be some overlap between the categories of sensitive data, they provide useful thematic groups and provide the additional advantage that, where applicable, they can allow for compatibility with existing regulation. These categories are either likely to (e.g., genetic, ethnicity, mental health, children and minors), or could conceivably (e.g., addictions and substance abuse, sexually-transmitted disease, disability, reproductive care, palliative care, geolocation), be present when sharing data sets for health-related genomic research.

### A Data Sharing Privacy Test

Having identified data categories which may contain information requiring a heightened level of protection in the context of health-related data, it was clear that for some of the areas considered some information within the categories would be more sensitive, e.g., ethnicity information about small or vulnerable population groups. We therefore developed a method for assessing different types of health-related data falling within these categories using a Data Sharing Privacy Test. We drew on the type of data privacy assessment the Treasury Board of Canada Secretariat proposed be conducted prior to contracting personal information.^7^ This is an ‘invasion-of-privacy’ test, which provides guidance in determining whether a contract would result in harm or injury to an individual by considering three main factors: the sensitivity of the information, the expectations of the individual, and the probability and gravity of injury.

In addition to variation in sensitivity within defined categories of sensitive data such as data concerning ethnicity, what constitutes sensitive data may be understood better from the perspective of their (mis)use, i.e., the privacy harms that could manifest, and to what magnitude, if these data were misappropriated or misused. This risk-based approach better aligns with the rationale of classifying data as sensitive. As the European Union’s expert committee on data protection issues (Article 29 Data Protection Working Party) explained: ‘The rationale behind regulating particular categories of data in a different way stems from the presumption that misuse of these data could have more severe consequences on the individual’s fundamental rights, such as the right to privacy and non-discrimination, than misuse of other, ‘normal’ personal data'.^8^ Thus, it could be said that *sensitive data are data whose improper use, including unwarranted disclosure, could reasonably be expected to cause serious physical or moral harm, significant financial loss or excessive personal distress to the data subject and/or related others*. These data include not only data which by their nature may reasonably be said to contain sensitive information, but also data from which sensitive information with regard to an individual or group can reasonably be abstracted or concluded. We therefore specifically consider the potential resulting harm that may result as a consequence of data re-identification as the second point of our Data Sharing Privacy Test.

The final point we propose considering with the Data Sharing Privacy Test is the expectations of the individuals concerned with respect to the sharing of these data. The information that has been provided to research participants and patients during the consent process may inform this assessment, as well as social studies of public perceptions of relevant risks and benefits. We note that where data sharing has been agreed to, there is an expectation that researchers will honour this consent.

**Figure 1:**
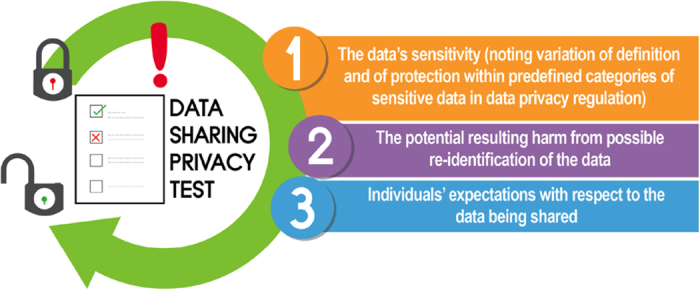
The three steps of a Data Sharing Privacy Test to distinguish degrees of data sensitivity within categories of data recognised as ‘sensitive’.

The Data Sharing Privacy Test (Figure 1) is therefore a privacy impact assessment based on:

1. The data’s sensitivity (noting variation of definition and of protection within predefined categories of sensitive data in data privacy regulation);
2. The potential resulting harm from possible re-identification of the data; and
3. Individuals’ expectations with respect to the data being shared.

All three points will depend to some extent on context, e.g., the jurisdiction of patients and research participants and applicable laws and socio-cultural values, as well as the views of individuals and communities. The first two points should nonetheless usually lead to similar classification of data from different individuals in the same environment, whereas the third point will often depend on individual preferences.

### Degrees of data sensitivity

We propose that some or all of the data in each sensitive data category (Table 1) may require a heightened level of protection as determined using our proposed Data Sharing Privacy Test.

Our approach of developing the categories based on legal analysis was not an attempt or a proposal to redefine sensitive data as a legal term, or to partition the term into ‘more sensitive’ and ‘less sensitive’ data. Rather, it was deemed a robust way for us to ensure that we had considered appropriate categories for a heightened degree of protection when providing access to data. A similar approach was previously proposed for the treatment of protected health information in medical records by the UK National Health Service with the creation of a ‘sealed envelope’ component of electronic health records which required a higher level of protection and could not be shared without patients’ explicit consent.^9^ While this approach for differential treatment of medical record information raised different issues for patients such as when emergency care teams should have access to certain information and the stigma of having a sealed envelope,^10^ it flowed from the common-sense understanding that not all health-related data are equally sensitive, and that the principle of proportionality should be applied in situations that engender both risk and potential benefit. As the Article 29 Data Protection Working Party of the European Union stated in a 2011 advice paper ‘[C]ategories of data display major differences in the degree of sensitivity. For example, health data may range from information about a simple cold to stigmatizing information about illnesses or disabilities. This leads to difficulties in practice, as the individual’s consent is required even for unproblematic processing of such data'.^8^ Data concerning health ranges from information that would be very unlikely to cause any person harm to stigmatizing information about present or future disease or disability. Thus, protection levels can range from *de minimus* to stringent, depending on sensitivity. Furthermore, the recently agreed EU General Data Protection Regulation proposal includes requirements for a data protection impact assessment to determine levels of risk in data processing, some of which may ultimately apply to genomic data sharing in scientific research and clinical care:

‘The likelihood and severity of the risk for the rights and freedoms of the data subject should be determined in function of the nature, scope, context and purposes of the data processing. Risk should be evaluated based on an objective assessment, by which it is established whether data processing operations involve a risk or a high risk.’^6^

For the purposes of the Beacon Project and similar data sharing scenarios, *health-related data should receive prima facie higher protection if it falls in one of the data categories presented here (Table 1), but only if on further analysis it falls in the zone of higher protection within these categories. *Of course, at all times, processing of personal data must respect relevant laws.

### Points-to-Consider

We developed further guidance for the types of health-related information that on face value would require higher protection within the categories of sensitive data identified in regulatory approaches, which is based on their perceived potential to lead to stigmatisation or discrimination, and on the vulnerability of data subjects and their privacy interests. Specifically, we provide ‘Points-to-Consider’ for such sensitive data, recognising that socio-economic and cultural factors are very important in their interpretation and that the exercise of professional judgment in particular contexts will play an important role. As per point 3 of the Data Sharing Privacy Test, patient and research participant consent will need to be considered along with this guidance, which, although informed by social science research into public views, including those of communities and other groups, does not account for individual privacy preferences that are known to vary considerably for the types of information we consider.^11^

Data falling in the following categories could include health-related data on symptoms, diagnosis and treatment (e.g., prescription medicines).

#### Ethnicity

Information about ethnicity or ethnic origin should receive higher protection if it concerns small or vulnerable groups. The determination of which groups are considered small or vulnerable must be done locally as this is affected by social and political context.^12^ Furthermore, analysis of this information in research raises important questions about how it has been recorded and the standardisation of terms describing groups.^13^ Using local publicly acceptable designations, such as those of national census categories, may reduce the risks for individuals. That research participants or patients belong to a particular population group should be carefully determined, especially for those groups that are considered small or vulnerable. The voluntary disclosure of such information makes individuals aware this information may be used for research or clinical care, and therefore more likely to consider potential consequences of its use for their communities. Group names should also be respectful of communities’ views and good practice has been to consult community members about how groups are described.^14^

#### Genetic

The category of genetic data could include biological filiation (e.g., paternity) or forensic identification data but we limit our discussion to information about clinical, genetic conditions.

In the context of the Beacon Project, this data category usually refers to information about symptomatic genetic conditions that will have been described, more or less precisely, for the purposes of research studies or clinical care. As much diversity exists within this category, we further propose a set of ‘Points-to-Consider’ that expand on our proposed Data Sharing Privacy Test, especially its first two points: (1) the sensitivity of the data, and (2) the potential resulting harm from possible re-identification of an individual’s data (Table 2).

First, if the genetic condition is outwardly visible, its sensitivity theoretically would be considered very high, but visible conditions are not private *per se*. Irrespective, the severity of the condition informs the assessment of potential resulting harm from data re-identification, which, in the absence of anti-discrimination protections, is likely to be greater for more severe conditions. Information about severe conditions should receive higher protection. However, agreeing on degrees of severity for genetic conditions is not without its challenges. Past research has demonstrated considerable disagreement between experienced genetics professionals as to what constitutes a ‘serious’ genetic condition.^15^ Genetics professionals also expressed a strong aversion to such classification as genetic conditions vary in expression and are perceived differently by individuals and in different cultures, to say nothing of the varying social and economic consequences this classification may have for patients. The American College of Medical Genetics and Genomics has nonetheless recently agreed on a list of genes in which pathogenic variants should be reported to patients undergoing clinical genome-scale sequencing if they wish to receive this information.^16,17^ The list was based on the likelihood of severe disease resulting from the variants and the possibility of its prevention. In contexts in which carrier status is recorded, assessments of severity should take variant penetrance and age of disease onset into account.

It should also be considered whether the genetic condition is associated with stigmatizing information such as information on mental health or disability, or if it has consequences for reproductive health. If associated with information that would fall in the higher zone of protection of one of the other categories of sensitive information identified here (Table 1), the genetic condition could be considered potentially stigmatizing information and receive higher protection.

For rare genetic conditions, two additional points should be considered. Does it reveal the likely ancestry or geographical location of individuals? If the rare disease is associated with a certain ancestry, is this information about ethnicity that may be considered potentially stigmatizing (see previous section)? If so, or if the rare disease provides information about likely geographical location of individuals, it should receive higher protection.^13^ Paradoxically, more not less sharing is required to elucidate the causes of rare diseases.^18^

Finally, the probability of carrier status for relatives should be considered along with disease penetrance and age of onset. Genetic conditions that are familial, highly penetrant, of early onset and cause serious disease should receive higher protection. If genetic conditions are not familial, the severity threshold for providing more protection may be higher and reliance on individual consent greater.

#### Mental health

All information pertaining to mental health should receive higher protection. This category is defined as information about any disturbance in an individual’s cognition, emotion regulation or behaviour that may reflect a dysfunction in the psychological, biological or developmental processes underlying mental functioning. This definition has been adapted to suit this context from the American Psychiatric Association Fifth Edition of the Diagnostic and Statistical Manual of Mental Disorders (DSM-5) definition of mental disorder^19^ with reference to the World Health Organization International Statistical Classification of Diseases and Related Health Problems 10th Revision (ICD-10).^20^

#### Addictions, substance abuse

All information relating to substance abuse and addictions should receive higher protection. It is reasonable, however, to exempt information about low levels of consumption of addictive substances, such as alcohol and tobacco, reflecting their use rather than abuse, thereby requiring a lower level of protection.

#### Sexually-transmitted disease

All information about sexually-transmitted disease should receive higher protection.

#### Children, minors

Data concerning children and minors require protection and special consideration by virtue of incapacity to meaningfully exercise and express their autonomous interests, and this beyond the general considerations of context that are standard for information in other data categories.^21^ Although studies show adolescents are more likely to withhold types of information that are generally considered sensitive by the broader population, such as sexual orientation, drug use, depression and suicidal thoughts,^22,23^ their privacy interests may be greater than those of adults. In a study by Cheng *et al.*,^24^ a quarter of adolescents reported that they would not seek care for health concerns if they thought their parents, friends or teachers might find out. Second, predictive information such as for late-onset conditions should receive higher protection. Finally, wherever consent may be relied on to differentiate between levels of protection for data within the other categories of sensitive information, this should not be applied to data from children and minors as they may not be able to express their consent autonomously. It should be remembered, however, that like other vulnerable populations, children and minors should not be excluded from research on the sole basis of this ‘vulnerability’ as, e.g., there are paediatric conditions that are exclusive to this group.^25^

#### Disability

All information concerning disability should receive higher protection. The International Classification of Functioning, Disability and Health (ICF) defines disability as ‘an umbrella term for impairments, activity limitations and participation restrictions. Disability is the interaction between individuals with a health condition (e.g., cerebral palsy, Down syndrome and depression) and personal and environmental factors (e.g., negative attitudes, inaccessible transportation and public buildings, and limited social supports)'.^26^ Usually, disability legislation covers actual discrimination based on visible disability and not discrimination that is the result of being perceived as already disabled, as may occur with predictive genetic data on a late-onset condition where the individual is perceived as already having the condition (see, however, the exemplary approach of the Americans with Disabilities Act).

#### Reproductive care

All information concerning reproductive care, including data about contraception and abortion, should receive higher protection.

#### Palliative care

The World Health Organization defines palliative care as ‘an approach that improves the quality of life of patients and their families facing the problem associated with life-threatening illness, through the prevention and relief of suffering by means of early identification and impeccable assessment and treatment of pain and other problems, physical, psychosocial and spiritual'. Sensitive information requiring a higher level of protection may arise in the context of end-of-life care. Information concerning religious affiliation, especially, should receive higher protection.

#### Geolocation

Information on geolocation within areas smaller than a state, province or county (e.g., postal code, community district) should receive higher protection. Geolocation combined with environmental, health and socio-economic data can adversely affect service provision and property values.^27^

#### Other categories of sensitive data

With the exception of data concerning sex life, the following categories, while listed in some laws, were not commonly found in the regulatory and policy instruments we consulted. While we expect they may sometimes be found in genomic health data sets, without further research on their meaning, we cannot determine which types of data within these categories may present in research and healthcare data sets, nor whether they may require higher protection:

- Sex life
- Behavioural profiles
- Domestic violence
- Finances

## Discussion

A major challenge in applying our Data Sharing Privacy Test in the health-related context is its dependence on context, and therefore its reliance on contextual information, and on public perceptions (the views and attitudes of individuals and communities). For much of the contextual information, we rely on studies of law and regulation, which while imperfect representations of situations around the world, denote a common consensus within a given nation, albeit somewhat static. Assessing changing public perceptions is then perhaps the greatest challenge, yet much better understanding and methods exist today than even a decade ago. That we are intent on further determining the ELSI status of these data, and believe our approach is feasible, rests on two main facts. First, the policy and design of current data sharing projects such as the Beacon Project allows sharing of health-related data even if they are categorized as requiring higher protection. This enables degrees of protection without large negative consequences for data access and use. Second, we believe the potential for application of guidance in the area of research, as well as in healthcare, is vast and may be beneficial.

### The Beacon Project

A recent study showed the theoretical confidence with which it would be possible to use the Beacon API to determine the presence or absence of an individual’s genetic data in a particular Beacon database.^28^ Our novel concept of ‘tiered protection’ will be piloted as a means to avoid the associated risk of inference of phenotypic information that would not otherwise be available from an individual’s genetic information, or of other health-related information that may be provided through Beacons, whenever such information falls within the zone of higher protection. This will be achieved by aggregating Beacons holding data that are linked to sensitive information requiring a higher level of protection, thereby reducing the probability that any one individual present in the data set is likely to have a particular trait or condition. As the Beacon Project develops new applications, it is also envisaged our method will be used to determine when user registration may be required to gain access to more detailed data through Beacons (Registered Access (Dyke *et al.*)^29^).

As well as refining the boundaries of the zone of higher protection, from which several tiers may emerge, our field-testing with scientific studies involved in the Beacon Project and with other Global Alliance projects may bring to light additional categories and types of data that require consideration. It will also allow us to gain a better understanding of the preponderance of more and less sensitive health-related data.

We have proposed a systematic approach to categorizing health-related data from genomic research and clinical data sets as to the level of protection required in the context of the Beacon Project and other projects foreseeing the sharing of health-related data. In clarifying and defining data categories requiring heightened protection, providing ‘Points-to-Consider’ for case-by-case assessment, and identifying data categories that require further consideration, we outline practical tiers of protection that can be used to implement responsible data sharing. We now seek to engage in broader community consultation, and to submit our Data Sharing Privacy Test to international perspectives, with a view to assessing the level of consensus on our approach. It is important to stress again that context matters as evidenced by the fact that generally the rare disease communities seek greater open and accessible data sharing even while presumably heightened protection would often apply to rare disease data. While we believe this guidance will be useful to implement a range of proportionate data governance and security mechanisms in other settings, due consideration of the circumstances of data collections (e.g., of their collection, storage and access) and of potential differences with the Beacon Project will be necessary as the tiers of protection we propose for this project may not be suitable to other situations.

## Materials and methods

To begin, we reviewed which categories of data were deemed sensitive in data privacy regulation and policy guidelines in Europe and in the United States.^8,30–32^ This led to the identification of 12 categories of information that could reasonably pertain to health-related data and which accordingly may warrant a heightened degree of protection. One other category was added to this list (palliative care), which was not considered in the regulatory and policy instruments we consulted, but was identified during consultation on the proposed categories with the international scientific community via the Global Alliance Data Working Group.^33^ Through consultation of the international data privacy law literature, we then conducted a more exhaustive review of the definitions of, or statements on, sensitive personal data in the major data privacy laws in jurisdictions around the world (covering >60 laws in 55 jurisdictions; see Supplementary Table S1).^34–41^ This research led to the identification of one additional category of sensitive data, namely data concerning disability. That so much had been covered through the initial study of EU Member States and US documents reflects the degree of international harmonisation of standards aligned with these nations.

## Figures and Tables

**Table 1:** List of categories of sensitive data identified in regulatory and policy data privacy instruments, and in consultation with the scientific community (see also Supplementary Table S1)

*Categories of sensitive data*
Ethnicity
Genetic
Mental health
Addictions, substance abuse
Sexually-transmitted disease
Children, minors
Disability
Reproductive care
Palliative care
Sex life
Behavioural profiles
Domestic violence
Geolocation
Finances

**Table 2:** Genetic data: Points-to-Consider for assessing the degree of sensitivity of data on clinical, genetic conditions

*Points-to-Consider*	
1	Is the genetic condition outwardly visible?
2	How severe is it? (serious disease, penetrance, age of onset)
3	Is it associated with what could be considered to be stigmatizing health information (e.g., associated with mental health, reproductive care, disability)?
4	Is it familial (i.e., potential carrier status/reproductive implications for family/relatives)?
5	Does it provide information about the likely geographical location of individuals?
6	Does it provide information about ethnicity that may be considered potentially stigmatizing information?
