# Supplementary material for: Sharing health-related data: a privacy test?
Source: NPJ Genom Med. 2016 Aug 17;1:16024–. doi: 10.1038/npjgenmed.2016.24 (PMC5158304; doi:10.1038/npjgenmed.2016.24)
Supplement: Supplementary Information [file npjgenmed201624-s1.pdf]

Supplementary Table S1. Table of data privacy laws in selected jurisdictions that define or have an explicit statement on sensitive personal data.

Country	Law	Definition of/Statement on sensitive personal data
Argentina	Personal Data Protection Law Number 25,326	Personal data revealing racial and ethnic origin, political opinions, religious, philosophical or moral beliefs, labor union membership, and information concerning health conditions or sexual habits or behavior.
Australia	Federal Privacy Act 1988	Information or an opinion about an individual's:<br>• racial or ethnic origin;<br>• political opinions;<br>• membership of a political association;<br>• religious beliefs or affiliations;<br>• philosophical beliefs;<br>• membership of a professional or trade association;<br>• membership of a trade union;<br>• sexual orientation or practices;<br>• criminal record that is also personal information;<br>• health information about an individual;<br>• genetic information about an individual that is not otherwise health information;<br>• biometric information that is to be used for the purpose of automated biometric verification or biometric identification; or<br>• biometric templates.
Austria	Data Protection Act, Federal Law Gazette part I No. 165/1999	Data relating to natural persons concerning their racial or ethnic origin, political opinion, trade-union membership, religious or philosophical beliefs, and data concerning health or sex life.
Belgium	Data Protection Act dated 8 December 1992	The Act distinguishes between three categories of sensitive personal data, for which distinct rules apply:<br>• personal data revealing a person's racial or ethnic origin, political opinions, religious or philosophical beliefs, sex life or trade union membership<br>• health-related personal data, and<br>• personal data relating to litigation that has been submitted to courts and tribunals as well as to administrative judicial bodies, relating to suspicions, prosecutions or convictions in matters of crime, administrative sanctions or security measures

Bulgaria	Law for the Protection of Personal Data, promulgated in the State Gazette No. 1 of 4 January 2002 (as amended)	Personal data revealing racial or ethnic origin; political, religious or philosophical beliefs; political, religious or philosophical convictions, membership in political parties or organizations, associations having religious, philosophical, political or trade-union goals or personal data which refer to health, sexual life or human genome.
Canada	Personal Information Protection and Electronic Documents Act (federal, private sector)	Not specifically defined.<br>"...Although some information (for example, medical records and income records) is almost always considered to be sensitive, any information can be sensitive, depending on the context. For example, the names and addresses of subscribers to a newsmagazine would generally not be considered sensitive information. However, the names and addresses of subscribers to some special-interest magazines might be considered sensitive." (Schedule 1, Principle 4.3.4)
	Personal Health Information Act (Manitoba)	Not specifically defined.<br>"...health information is personal and sensitive and its confidentiality must be protected so that individuals are not afraid to seek health care or to disclose sensitive information to health professionals." (Preamble)
Chile	Law 19,628 "On the protection of private life"	Personal data which refer to physical or moral characteristics of persons or facts or circumstances of their privacy or intimacy, such as personal habits, racial origin, ideologies and political opinions, beliefs or religious beliefs, states of physical or mental health and sexual life.
China	National Standard of Information Security Technology – Guideline for Personal Information Protection within Information System for Public and Commercial Services (Promulgated 05 Nov 2012 and effective on 01 Feb 2013, GB/Z 28828-2012	Personal information that, once it is leaked or altered, may bring about harmful influence to the subject of the indicated personal information. The concrete content sensitive personal information in different sectors is to be determined on the basis of the wishes of the subject of the personal information who receives the service and the particular characteristics of different sectors. Personal information listed as sensitive may include identity card numbers, mobile telephone numbers, ethnicity, political viewpoints, religious beliefs, genes, fingerprints, etc.
Colombia	Law 1581 and article 3 of Decree 1377	Data that affect the intimacy of the data owner, or whose improper use could lead to discrimination, such as data revealing racial or ethnic origin, political orientation, religious or

		philosophical beliefs, trade union membership, social organizations, human rights organizations, or those organizations that promote the interests of any political party or that ensure the rights and guarantees of opposition political parties, as well as data relating to health, sexual life and biometrics.
Costa Rica	Law No. 8968, Protection in the Handling of the Personal Data of Individuals	Personal information relating to ideological orientation, creed, sexual preferences.
Cyprus	Processing of Personal Data (Protection of the Individual) Law of 2001	Data concerning racial or ethnic origin, political convictions, religious or philosophical beliefs, participation in a body, association and trade union, health, sex life and erotic orientation as well as data relevant to criminal prosecutions or convictions.
Czech Republic	Act no. 101/2000 Coll., on the Protection of Personal Data	Personal data revealing nationality, racial or ethnic origin, political attitudes, trade union membership, religious and philosophical beliefs, conviction of a criminal act, health status and sexual life of the data subject, as well as any genetic data of the data subject or biometric data permitting direct identification or authentication of the data subject.
Denmark	Act on Processing of Personal Data (2000)	Personal data revealing racial or ethnic origin, political opinions, religious or philosophical beliefs, trade union membership, or data concerning health or sex life.
Finland	Personal Data Act 523/1999	Personal data that relates to or is intended to relate to racial or ethnic origin; the social, political or religious affiliation or trade union membership of a person; criminal act, punishment or other criminal sanction; the state of health, illness or handicap of a person or the treatment or other comparable measures directed at the person; the sexual preferences or sex life of a person; or the social welfare needs of a person or the benefits, support or other social welfare assistance received by the person.
France	Law No. 78 17 of 6 January 1978 on 'Information Technology, Data Files and Civil Liberty'	Personal data that reveals directly or indirectly, racial and ethnic origins, political, philosophical or religious opinions or trade union affiliation of persons, or which concern their health or sexual life.
Germany	Federal Data Protection Act	Any information on racial or ethnic origin, political opinions, religious or philosophical beliefs, trade union membership, health or sex life.
Greece	Law 2472/1997 on the Protection of Individuals with	Data referring to racial or ethnic origin, political opinions, religious or philosophical beliefs,

	regard to the Processing of Personal Data	membership of a trade union, health, social welfare and sex life, criminal charges or convictions as well as membership to societies dealing with the aforementioned areas.
Honduras	Law for Transparency and for Access to Public Information (Article 3.5, Decree 170-2006)	Those personal data relating to ethnic or racial origin, physical, moral or emotional characteristics, home address, telephone number, personal electronic address, political participation and ideology, religious or philosophical beliefs, health, physical or mental status, personal and familiar heritage and any other information related to the honour, personal or family privacy, and self-image.
Hungary	Act No. CXII of 2011 on Informational Self Determination and Freedom of Information	Personal data revealing racial or national origin, political opinion or party membership, religious or other philosophical belief, membership in an interest representation organization ,or sex life; and personal data concerning health, addiction, or criminal personal data.
Iceland	Act No 77/2000 on the Protection and Processing of Personal Data	Data on origin, skin colour, race, political opinions, religious beliefs and other life philosophies; data on whether a man has been suspected of, indicted for, prosecuted for or convicted of a punishable offence; health data, including genetic data and data on use of alcohol, medical drugs and narcotics; data concerning sex life (and sexual behaviour); and data on trade union membership.
India	There is no specific legislation on privacy and data protection in India. Information Technology (Reasonable Security Practices and Procedures and Sensitive Personal Data or Information) Rules (IT Ministry) (applies to corporate entities collecting, processing and storing personal data)	Information relating to: password; financial information e.g. bank account/credit or debit card or other payment instrument details; physical, physiological and mental health condition; sexual orientation; medical records and history; biometric information.<br>Any information that is freely available in the public domain is exempt from the above definition.
Ireland	Data Protection Act 1988	Personal data as to racial origin, political opinions, religious or other beliefs, physical or mental health, sexual life or criminal convictions.
Israel	Protection of Privacy Law, 5741-1981	Data on the personality, intimate affairs, state of health, economic position, opinions and beliefs of a person; and other information if designated as such by the Minister of Justice with the approval of the Constitution, Law and Justice Committee of the Knesset. [Note: No such

		determination has been made to date.]
Italy	Legislative Decree no. 196 of 30 June 2003	Personal data allowing the disclosure of racial or ethnic origin, religious, philosophical or other beliefs, political opinions, membership of parties, trade unions, associations or organizations of a religious, philosophical, political or trade unionist character, as well as personal data disclosing health and sex life.
Japan	Protection of Personal Information Act (2015)	Personal information that contains descriptions that have been specified by Cabinet Order to require special consideration in handling so as to avoid any unfair discrimination, prejudice or other disadvantage to an individual based on person's race, creed, social status, medical history, criminal records or the fact that a person has incurred damages through an offense, etc.
Lithuania	Law on Legal Protection of Personal Data (1996)	Data concerning racial or ethnic origin of a natural person, his political opinions or religious, philosophical or other beliefs, membership in trade unions, and his health, sexual life and criminal convictions.
Luxembourg	Protection of Persons with regard to the Processing of Personal Data (2002)	Personal Data relating to racial or ethnic origin, political opinions, religious or philosophical beliefs, trade union membership, and the health or sex life, including the processing of genetic data.
Macau	Macau personal data protection Law no. 8/2005 of August 22nd	Any personal data revealing political persuasion or philosophical beliefs, political and join trade unions affiliation, religion, private life and racial or ethnical origin as well as data related to health or sex life, including genetic data.
Malaysia	Personal Data Protection Act 2010	Any personal data consisting of information as to the physical or mental health or condition of a data subject, his political opinions, his religious beliefs or other beliefs of a similar nature, the commission or alleged commission by him of any offence or any other personal data as the Minister of Information, Communications and Culture (‘Minister’) may determine by order published in the *Gazette*.
Malta	Data Protection Act (Chapter 440 of the Laws of Malta)	Personal data that reveals race or ethnic origin, political opinions, religious or philosophical beliefs, membership of a trade union, health, or sex life.
Mauritius	Data Protection Act 2004	Personal information concerning a data subject that include information as to racial or ethnic origin; political opinion or adherence; religious belief or other belief of a similar nature; membership to a trade union; physical or mental health, sexual preferences or practices; the

		commission or alleged commission of an offence; or any proceedings for an offence committed or alleged to have been committed by him, the disposal of such proceedings or the sentence of any court in such proceedings.
Mexico	Federal Law on Protection of Personal Data held by Private Parties	Personal data touching on the most intimate areas of the data subject's life, or data the misuse of which may lead to discrimination or serious risk to the data subject. Specifically, the definition includes data which may reveal items such as racial or ethnic origin, present or future health status, genetic information, religious, philosophical or moral beliefs, union affiliation, political views, and sexual preference.
Monaco	Data Protection Law n° 1.165 of 23 December 1993	Personal data which is likely to reveal racial or ethnic origin, political opinions, religious or philosophical beliefs, trade union membership, or data concerning health, including genetic data, and data pertaining to sex life, lifestyle or social welfare measure.
Morocco	Law n° 09-08 of 18 February 2009 relating to the protection of individuals with respect to the processing of personal data	Any information pertaining to a concerned individual that reveals racial and ethnic origin, political, philosophical, religious opinions or trade union affiliation, or that concern sex life or health, including genetic data.
Netherlands	Personal Data Protection Act	Personal data regarding a person's religion or philosophy of life, race, political persuasion, health and sexual life, trade union membership, criminal behaviour and personal data regarding unlawful or objectionable conduct connected with a ban imposed as a result of such conduct.
Norway	Personal Data Act (LOV-2000-04-14-31)	Information relating to racial or ethnic origin, or political opinions, philosophical or religious beliefs; the fact that a person has been suspected of, charged with, indicted for or convicted of a criminal act; health conditions; sex life; or membership in unions.
Peru	Personal Data Protection Law No. 29733	Personal data consisting of biometric data; racial and ethnic origin; income; political, religious, philosophical or moral opinions or convictions; personal habits; union membership; and data related to health or sexual life.
Philippines	Data Privacy Act of 2012	Personal information:<br>• about an individual's race, ethnic origin, marital status, age, color, and religious, philosophical or political affiliations;<br>• about an individual's health, education, genetic or sexual life of a person, or to any proceeding for any offense committed or alleged to have been

		committed by such person, the disposal of such proceedings, or the sentence of any court in such proceedings;<br>• issued by government agencies peculiar to an individual which includes, but not limited to, social security numbers, previous or current health records, licenses or its denials, suspension or revocation, and tax returns; and<br>• specifically established by an executive order or an act of Congress to be kept classified.
Poland	Personal Data Protection Act of 29 August 1997	Personal data revealing racial or ethnic origin, political opinions, religious or philosophical beliefs, religious, party or trade union membership, as well as personal data concerning health, genetic code, addictions or sex life and data relating to convictions, decisions on penalty, fines and other decisions issued in court or administrative proceedings.
Portugal	Portuguese Data Protection Law – Law nº. 67/98, of October 26th	Personal data revealing one's philosophical or political beliefs, political affiliations or trade union membership, religion, private life and racial or ethnic origin, and also data concerning health or sex life, including genetic data.
Romania	Law no 677/2001 on the protection of individuals with regards to the processing of personal data and the free movement of such data	Personal data regarding racial or ethnical origin, political, religious or philosophical beliefs or those of similar nature, trade union allegience, and personal data regarding the state of health or sex life.
Russia	Data Protection Act No. 152 FZ dated 27 July 2006	Data related to race, national identity, political opinions, religious and philosophical beliefs, health and private life, and biometric data.
Serbia	Law on Personal Data Protection	Data relating to ethnicity, race, gender, language, religion, political party affiliation, trade union membership, health status, receipt of social support, victims of violence, criminal record and sexual life.
Slovak Republic	Act No. 428/2002 Coll. on the Protection of Personal Data	Personal data revealing racial or ethnic origin, political opinions, religious or philosophical beliefs, membership in political parties or movements, trade union membership, and the processing of data concerning health or sex life.
South Africa	Protection of Personal Information Act, 2013	Personal information concerning a person's religious or philosophical beliefs, race or ethnic origin, trade union membership, political persuasion, health or sex life or biometric information, or criminal behaviour to the extent that such information relates to (i) the alleged commission by a data subject of any offence; or

		(ii) any proceedings in respect of any offence allegedly committed by a data subject or the disposal of such proceedings.
South Korea	Personal Information Protection Act (PIPA)	Personal information including ideology, belief, admission/exit to and from trade unions or political parties, political mindset, health, sexual life, and other personal information which is likely doing harm to privacy of data subjects. The Enforcement Decree of PIPA includes DNA information acquired from a genetic examination and criminal history data.
Spain	Special Data Protection Act 1999	Personal data related to political orientation, religion, beliefs, trade union membership, ethnic origin, health and sex life.
Sweden	Personal Data Act, SFS 1998:204	Personal data that reveals race or ethnic origin, political opinions, religious or philosophical beliefs, and membership of trade unions, and personal data relating to health or sex life.
Switzerland	Federal Act on Data Protection of 19 June 1992	Personal data on:<br>• religious, ideological, political or trade union related views or activities<br>• health, the intimate sphere or racial origin<br>• social security measures, and<br>• administrative or criminal proceedings and sanctions.<br>'Personality profiles' are also treated as sensitive personal data. Personality profiles are collections of data that allow the appraisal of essential characteristics of the personality of an individual.
Taiwan	Personal Information Protection Act	Personal information of medical treatment, genetic information, sexual life, health examination and criminal record.
Trinidad and Tobago	The Data Protection Act, 2011	Personal information on a person's:<br>• racial or ethnic origins<br>• political affiliations or trade union membership<br>• religious beliefs or other beliefs of a similar nature<br>• physical or mental health or condition<br>• sexual orientation or sexual life, or<br>• criminal or financial record.
UAE-Dubai (Dubai International Financial Centre)	DIFC Law No. 1 of 2007 Data Protection Law	Personal data revealing or concerning (directly or indirectly) racial or ethnic origin, communal origin, political affiliations or opinions, religious or philosophical beliefs, criminal record, trade union membership and health or sex life.
Ukraine	Law of Ukraine No. 2297 VI (on Protection of Personal	Personal data about racial or ethnic origin, political views, religious or other convictions,

	Data)	membership in political parties and trade unions, criminal charges or convictions as well as data with regard to health or sexual life.
United Kingdom	Data Protection Act 1998	Personal data consisting of information as to:<br>• the racial or ethnic origin of the data subject<br>• his political opinions<br>• his religious beliefs or other beliefs of a similar nature<br>• whether he is a member of a trade union<br>• his physical or mental health or condition<br>• his sexual life<br>• the commission or alleged commission by him of any offence, or<br>• any proceedings for any offence committed or alleged to have been committed by him, the disposal of such proceedings or the sentence of any court in such proceedings.
United States	No comprehensive data protection legislation. There are approximately 20 sector-specific or medium-specific privacy laws, and hundreds of such laws among the 50 states. The scope of the laws is generally limited to the practices of specific industries. These laws tend to protect consumers from specific types of privacy harms.	Varies widely by sector and by type of statute. Generally sensitive data applies to personal health data (e.g. HIPAA), credit worthiness data, personal data collected online from children under 13, and data that can be used to carry out identity theft or fraud.
Uruguay	Data Protection Act Law No. 18.331 (11 August 2008); Decree No. 414/009 (31 August 2009)	Any kind of personal data evidencing: racial or ethnic origin, political preferences, religious or moral beliefs, trade union membership and any kind of information concerning health or sexual life.
